# Supplementary material for: Elucidating the Mesocarp Drupe Transcriptome of Açai (Euterpe oleracea Mart.): An Amazonian Tree Palm Producer of Bioactive Compounds
Source: Int J Mol Sci. 2023 May 26;24(11):9315. doi: 10.3390/ijms24119315 (PMC10253617; doi:10.3390/ijms24119315)
Supplement: Supplementary file 1 [file ijms-24-09315-s001.zip › Additional files.pdf]

Additional files

Elucidating The Mesocarp Drupe Transcriptome of Açaí (*Euterpe oleracea* Mart.), An Amazonian Palm Tree Producer of Bioactive Compounds.

Elaine dos Santos, Hubert Schaller, Artur Silva, Hervé Rogez, Sylvain Darnet*

***Correspondence:** Correspondence: Sylvain Darnet shd@laposte.net

Additional file S1: **Table S1.** Quality and completeness statistics of *Euterpe oleracea* transcriptomes obtained during *de novo* assembly optimization. **(A)** Single *k*-mer results. **(B)** Multiple *k*-mer additive approaches. Statistics were calculated using BUSCO pipeline with plant section and Transrate tool with *Phoenix dactylifera* proteome. Read coverage and mapping statistics were obtained using bowtie, SAMtools, and in-house shell scripts. **Table S2.** *Euterpe oleracea* transcripts with functional annotation retrieved from Blast2GO. UniProt database, section *Viridiplantae*, was used to performed BLASTX search with e-value of 1e-3. **Table S3.** Gene Ontology enrichment terms in genes restricted to *Euterpe oleracea*. Gene list restricted to *Euterpe oleracea* is based on singleton category in OMA pipeline. The GO enrichment test was based on Fisher-test performed in Blast2GO, comparing the list of restricted genes and all genes predicted in the transcriptome. An FDR of 5% was considered as the cut-off for enrichment. **Table S4.** Genes of *Euterpe oleracea* with positive selection. The detection was based on all orthologs groups present as single-copy in the *Euterpe oleracea, Phoenix dactylifera*, *Arabidopsis thaliana* and *Elaeis guineensis*. The POTION pipeline was used to detect the positive selection with site-models from CodeML/PAML. **Table S5.** Genes from anthocyanin pathway in *Arabidopsis thaliana, Vitis vinifera, Solanum lycopersicum, Euterpe oleracea, Phoenix dactylifera* and *Elaeis guineensis*. All orthologs and paralogs of *Arabidopsis thaliana* genes associated with anthocyanin pathways were retrieved using OMA pipeline. Splicing isoforms are marked with an X symbol. **Table S6.** Genes from tocopherol pathway in *Arabidopsis thaliana, Vitis vinifera, Solanum*

lycopersicum, Euterpe oleracea, Phoenix dactylifera and *Elaeis guineensis*. All orthologs and paralogs of genes associated with anthocyanin pathways were retrieved using OMA pipeline. Splicing isoforms are marked with an X symbol. **Table S7.** EST-SSRs predicted in *Euterpe oleracea* transcriptome and inter-species transferability to *Phoenix dactylifera* and *Elaeis guineensis*. (**A**) List of all SSRs positions detected by MISA tool. (**B**) Primer pair list designed by Primer3 to amplify SSRs with the flanking sequence in the transcripts. (**C**) List of SSRs found in the three palm trees. **Table S8.** Transcript annotation and classification in OMA groups. The orthology inference was performed using OMA and proteome predicted in *Arabidopsis thaliana, Vitis vinifera, Solanum lycopersicum, Euterpe oleracea, Phoenix dactylifera* and *Elaeis guineensis*. For each transcript, the classification in OMA groups, i.e., Hierarchical group (HOG_group column) and Orthologous group (OMA_group column; one-to-one ortholog), was reported. The other information is about the transcript, as organism origin (species), accession number in UniProt (access_number_protein), copy number in HOG group (species and copy_number in HOG, multicopy by species columns), the annotation in UniProt database (description column).

Additional file S2: **Figure S1.** Palm tree and fruit picture of açaí (*Euterpe oleracea*). (**a**) The multi-stemmed tree palm of açaí (*E. oleracea*). (**b**) Ripe black açaí bunch. (**c-f**) Four stages of maturation of black and (**g-j**) variety white *E. oleracea* fruits. (**c**) Green fruits, (**d**) fruits at the beginning of maturation, (**e**) black fruits and (**f**) black fruits covered with a wax cuticle. (**g**) Unripe white açaí fruit, (**h**) greenish yellow fruits, (**i**) mature fruits and (**j**) greenish yellow fruits covered with a wax cuticle. **Figure S2.** KEGG annotation of *Euterpe oleracea* transcriptome (**a**) Global view of classification; (**b**) categories found in metabolism; (**c**) Top 10 from “Biosynthesis of other secondary metabolites”

category. All y-axis is in transcript count. **Figure S3.** Distribution of EST-SSRs predicted in *Euterpe oleracea* transcriptome.

Additional file S3: **Data S1.** Orthoxml file with Hierarchical Groups. The inference was predicted by OMA tool and based on proteome comparison among species *Arabidopsis thaliana, Vitis vinifera, Solanum lycopersicum, Euterpe oleracea, Phoenix dactylifera* and *Elaeis guineensis*. **Data S2.** Orthoxml file with Orthologous Groups. The inference was predicted by OMA tool and based on proteome comparison among species *Arabidopsis thaliana, Vitis vinifera, Solanum lycopersicum, Euterpe oleracea, Phoenix dactylifera* and *Elaeis guineensis*.
