# Supplementary figures and images for: Elucidating the Mesocarp Drupe Transcriptome of Açai (Euterpe oleracea Mart.): An Amazonian Tree Palm Producer of Bioactive Compounds
Source: Int J Mol Sci. 2023 May 26;24(11):9315. doi: 10.3390/ijms24119315 (PMC10253617; doi:10.3390/ijms24119315)

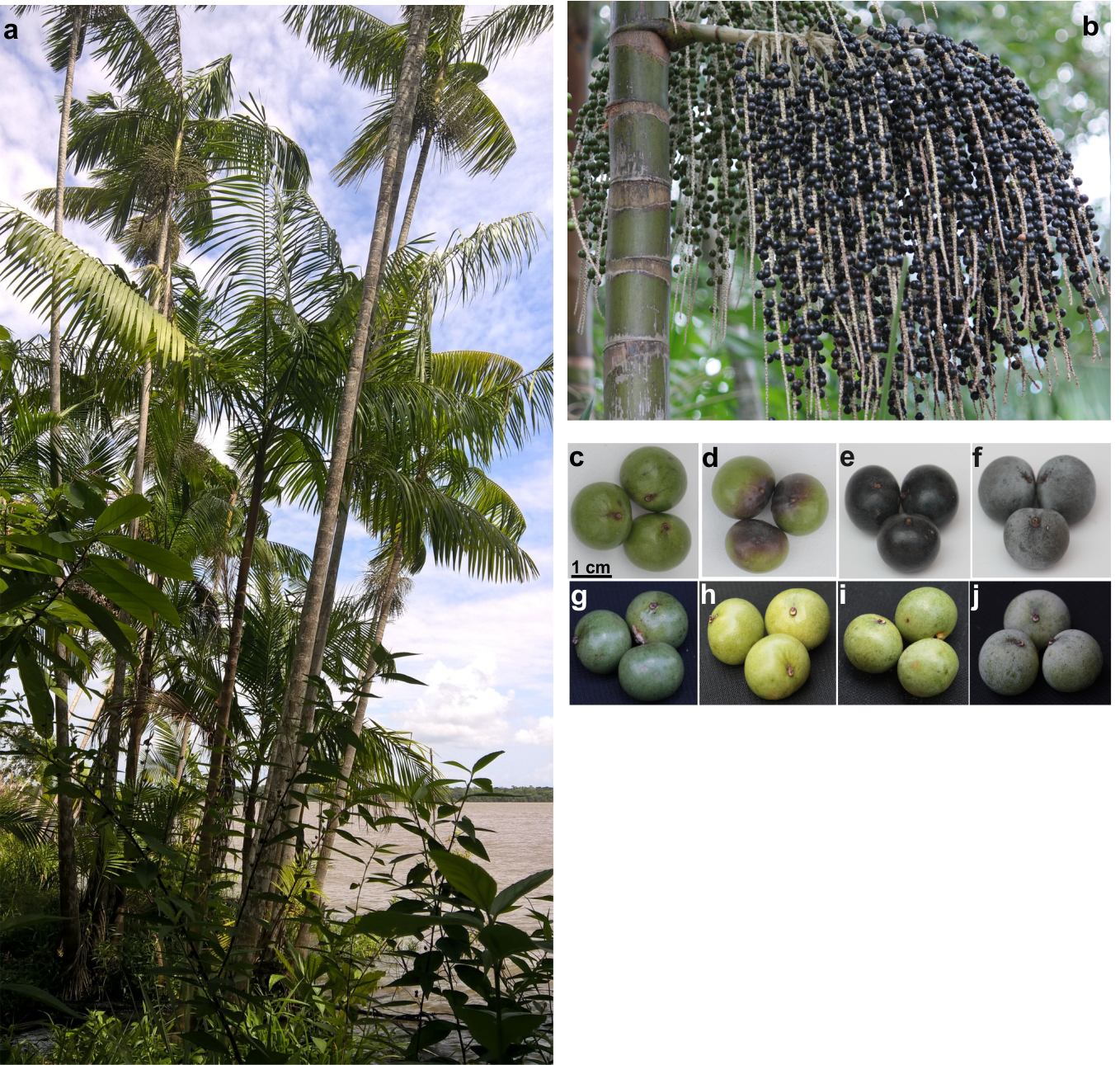

Supplement: Supplementary file 1 [file ijms-24-09315-s001.zip › Additional file S2_Figure S1.tif]

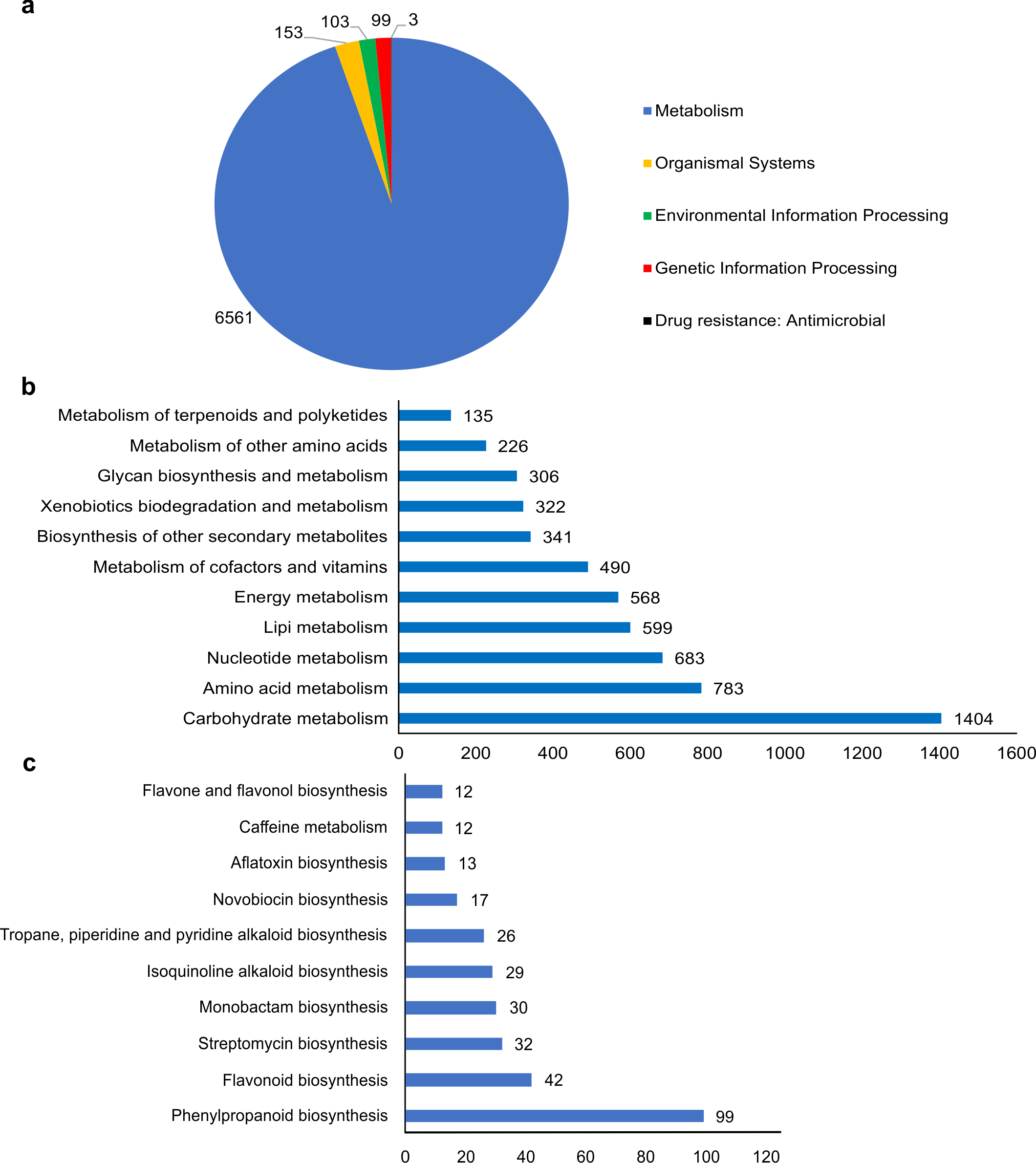

Supplement: Supplementary file 1 [file ijms-24-09315-s001.zip › Additional file S2_Figure S2.tif]

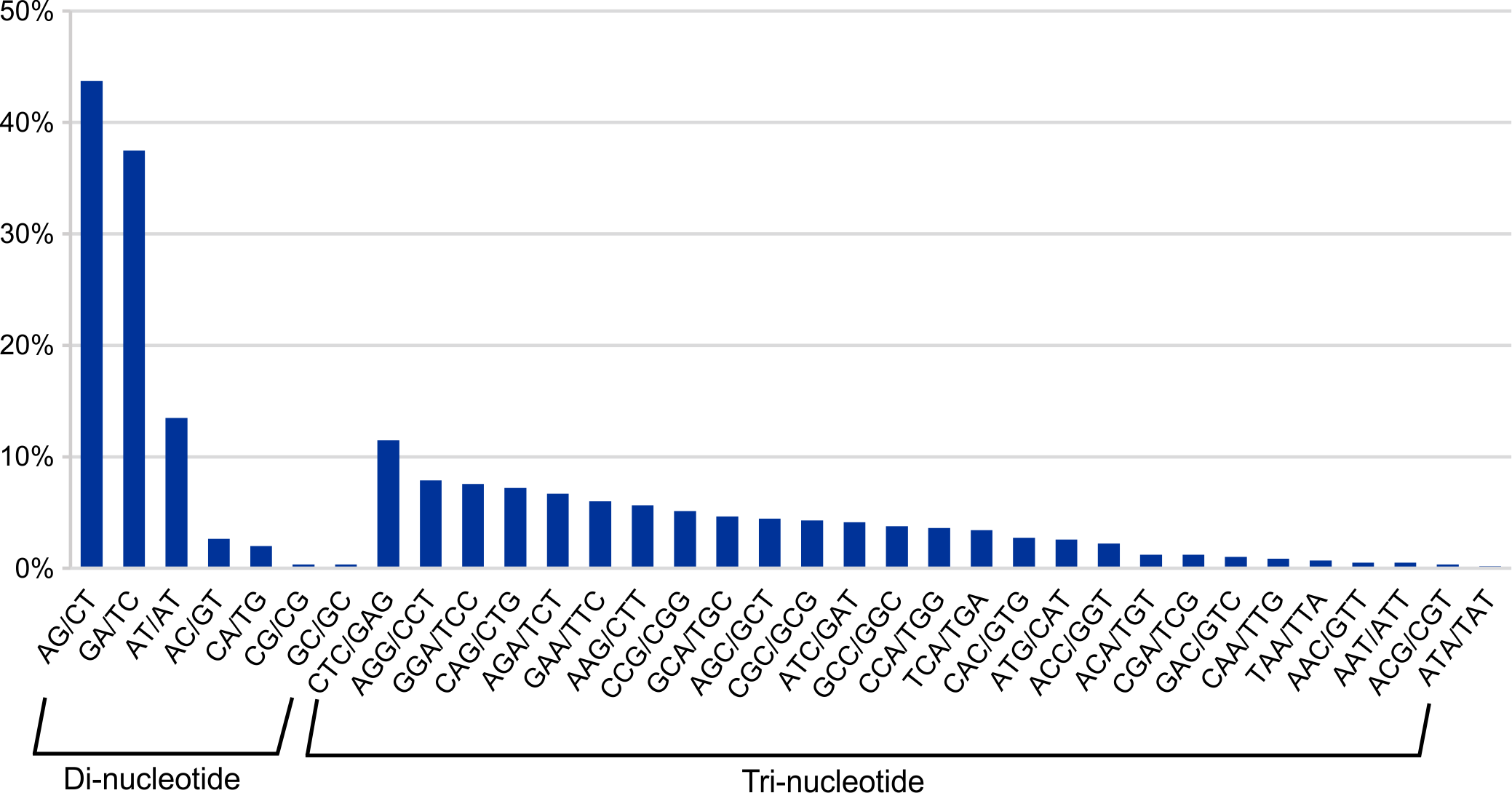

Supplement: Supplementary file 1 [file ijms-24-09315-s001.zip › Additional file S2_Figure S3.tif]
